# Text-fading based training leads to transfer effects on children's sentence reading fluency

**DOI:** 10.3389/fpsyg.2015.00119

**Published:** 2015-02-10

**Authors:** Telse Nagler, Sebastian P. Korinth, Janosch Linkersdörfer, Jan Lonnemann, Björn Rump, Marcus Hasselhorn, Sven Lindberg

**Affiliations:** ^1^Department of Education and Human Development, German Institute for International Educational Research (DIPF)Frankfurt am Main, Germany; ^2^Center for Individual Development and Adaptive Education of Children at Risk (IDeA)Frankfurt am Main, Germany; ^3^Department of Psychology, Goethe University Frankfurt am MainFrankfurt am Main, Germany

**Keywords:** reading rate, reading comprehension, training, transfer, intervention

## Abstract

Previous studies used a text-fading procedure as a training tool with the goal to increase silent reading fluency (i.e., proficient reading rate and comprehension). In recently published studies, this procedure resulted in lasting reading enhancements for adult and adolescent research samples. However, studies working with children reported mixed results. While reading rate improvements were observable for Dutch reading children in a text-fading training study, reading fluency improvements in standardized reading tests post-training attributable to the fading manipulation were not detectable. These results raise the question of whether text-fading training is not effective for children or whether research design issues have concealed possible transfer effects. Hence, the present study sought to investigate possible transfer effects resulting from a text-fading based reading training program, using a modified research design. Over a period of 3 weeks, two groups of German third-graders read sentences either with an adaptive text-fading procedure or at their self-paced reading rate. A standardized test measuring reading fluency at the word, sentence, and text level was conducted pre- and post-training. Text level reading fluency improved for both groups equally. Post-training gains at the word level were found for the text-fading group, however, no significant interaction between groups was revealed for word reading fluency. Sentence level reading fluency gains were found for the text-fading group, which significantly differed from the group of children reading at their self-paced reading routine. These findings provide evidence for the efficacy of text-fading as a training method for sentence reading fluency improvement also for children.

## Introduction

Efficient learning demands the ability to read texts at appropriate levels of accuracy and speed (Chard et al., 2002). Only if decoding is relatively effortless, accurate and fluent (Wolf and Katzir-Cohen, 2001), it is possible to free cognitive resources for higher-level demands, such as reading comprehension (Perfetti, 1985). The concept of reading fluency, however, is complex with several definitions focusing on different sub-aspects (Hudson et al., 2009). Oral reading fluency is defined as the ability to read text with speed, accuracy, and proper expression (National Reading Panel, 2000; Kuhn et al., 2010). Oral reading is often used to assess fluency in school or in research settings (Ridel, 2007), as it offers readily observable behavior (Price et al., 2012). Only few studies differentiate between oral and silent reading, arguing that even though the underlying processes have a lot in common and are interrelated, they can be distinguished as different skills (Share, 2008; Kim et al., 2011). Considering that skilled child and adult readers rarely engage in oral reading (Price et al., 2012) and classroom instruction primarily require silent and not oral reading (O'Connor et al., 2007), silent reading fluency (i.e., proficient reading rate and comprehension) seems especially important for independent learning (Bowey and Muller, 2005).

A comprehensive definition of reading fluency also comprises a distiction between different levels of reading material, for instance sublexical, lexical and connected text levels (Berninger et al., 2002; Klauda and Guthrie, 2008; Hudson et al., 2009). Single word reading fluency, for example, is mostly associated with lower level processing, such as orthographic and phonological processing and identification (Wolf and Katzir-Cohen, 2001). The more demanding the reading task, the more context and higher level processing skills are required. Hence, connected text reading fluency is assumed to comprise word-level reading skills as well as language processing and comprehension skills (Wolf and Katzir-Cohen, 2001; Jenkins et al., 2003a,b). It has been shown that reading fluency on the single word level (e.g., list reading) is highly correlated with connected text measures (Perfetti and Hogaboam, 1975). However, ongoing comprehension processes interact bi-directionally with reading fluency during sentence or text reading, while list reading primarily demands good decoding skills (Klauda and Guthrie, 2008). Considering the importance of silent reading fluency for self-instructed learning, we aim at expanding the research on silent reading fluency. More precisely we focus on silent reading fluency on the word, sentence and text level. With regard to previous research findings, showing that slow reading rates often result in reading comprehension deficits (Perfetti, 1985; Jenkins et al., 2003b), we investigate the question whether it is possible to foster silent reading fluency through training.

So far, several attempts were made to foster oral reading fluency through training; one of the most accepted methods being *repeated reading* (Hintikka et al., 2008). In this approach, children read the same material repeatedly while receiving feedback and corrections from peers or teachers (O'Connor et al., 2007). In *paired reading*, a similar approach, students are assigned to tutors (i.e., peers or adults) and instructed to read passages together using different strategies (e.g., reading at the same time; rereading sentences after the tutor read them aloud; Huemer et al., 2008). As a result, children can improve their reading fluency by adopting a more efficient reading rate with the help of a role model. Although training outcomes from these approaches indicated positive effects on reading rate and comprehension (Chard et al., 2002; O'Connor et al., 2007), a number of concerns were raised. Kuhn and Stahl (2003), for example, argued that effects observed in repeated reading studies might result from the increased amount of reading rather than from the repetition itself. It seems that overlearning solely fosters the reading rate for trained material, meaning that the reading enhancement cannot be transferred to unfamiliar texts or words (van Daal and van der Leij, 1992; Thaler et al., 2004; Berends and Reitsma, 2006). The focus on oral reading fluency renders the abovementioned training approaches time and personnel consuming, difficult to implement in classroom settings, and therefore economically unsatisfactory. Furthermore, focusing on oral reading fluency does not correspond to a call of the National Reading Panel (2000), which noted that the role of independent, silent reading should be investigated in more detail.

Hence, aiming at increasing individual's silent reading rates, a computerized training program was designed, which implemented a text-fading procedure manipulating the rate at which presented reading material appears on the computer screen (Breznitz and Nevat, 2006). For this manipulation, the individual reading rate is measured in a pretest, in which sentences are read in a self-paced manner. The average reading rate (i.e., milliseconds per letter) shown in the pretest is used as the baseline value for the per letter fading rate. At the start of the text-fading training, the presented sentences will therefore be faded out letter by letter in reading direction (i.e., in German from left to right) based on the individual reading rate, and this fading rate will be raised adaptively over the course of the training. To ensure that the text-fading procedure is adapted to individual needs, the fading rates of the text-fading training are frequently adjusted based on the answers to multiple-choice comprehension questions, which are presented after each sentence. Hence, if the individual exceeds a certain criterion (e.g., more than 80% correctly answered multiple choice questions in a determined number of trials) the fading rate will be accelerated for the next set of trials. If the criterion is met, the fading rate will be maintained; if the performance is below the criterion, the fading rate will be reduced. This adaptive procedure is designed to prevent cognitive overload as the fading rate remains in the individual's performance range. Each training item (i.e., sentence and comprehension question) is presented only once during the training to avoid overlearning.

The intention of the text-fading procedure is to prompt participants to read faster than they would normally in a self-paced manner (Breznitz and Berman, 2003) as the text-fading manipulation is assumed to generate a subjective feeling of time constraint (Nagler et al., 2014). Since increased reading speed is associated with an increase in reading comprehension (Huemer et al., 2008), the faster reading rates induced by the text-fading procedure are supposed to result in better reading comprehension performance. Automaticity theories (LaBerge and Samuels, 1974; Perfetti, 1985) may serve as the theoretical foundation for elicited gains in reading rate and comprehension (i.e., reading fluency) through the text-fading manipulation: A faster and more fluent reading process allows the release of cognitive resources (such as attention or working memory) and supposedly facilitates the allocation of cognitive capacity to higher cognitive functions, such as reading comprehension (Jenkins et al., 2003b). Hence, as discussed in previous studies using the text-fading procedure, the faster reading rates induced by time constraint might result in attention improvements (Breznitz, 1988), in a possible increase of available working memory capacity (Breznitz and Share, 1992; Breznitz, 1997a), or a shift from slow phonological to faster orthographical processing (Breznitz, 1987, 1997b). Furthermore, it was suggested that the subjective feeling of time constraint induced by the text-fading manipulation may trigger the individual to engage more frequently in direct fact retrieval (Nagler et al., 2014).

Only a small number of studies have investigated the effect of the text-fading training method so far. For a sample of adult native readers of Hebrew with and without reading difficulties, Breznitz et al. (2013) compared reading fluency of a text-fading group with a group who read the same sentences and answered the same questions, but at their own reading rate (i.e., self-paced group). It was demonstrated that participants receiving text-fading training improved their reading fluency expressed by higher per-letter reading rates as well as reading comprehension scores, both during training and at a later follow-up assessment, while self-paced reading controls did not. Similar results were reported for Hebrew speaking adolescents with and without reading difficulties, who were able to increase their reading speed and decrease reading errors after text-fading training. Furthermore, performance gains for participants with reading difficulties were significantly larger than those of normally achieving peers (Horowitz-Kraus and Breznitz, 2014).

The few studies focusing on text-fading training applied to reading impaired and normally achieving children produced somewhat diverse results (Snellings et al., 2009; Paige, 2011; Berninger et al., 2013). Focusing on the comparison of established training programs to text-fading training, Berninger et al. (2013) and Paige (2011) investigated training outcomes in samples of English-speaking dyslexic children. Results indicated positive effects on reading performance (e.g., phonological decoding, word identification accuracy) through text-fading, which however, did not differ from gains of the compared training approaches. Furthermore, no specific measures regarding reading fluency on different levels (such as word, sentence, text) were applied after training in these studies.

Snellings et al. (2009) applied a design in which children completed either a text-fading or a self-paced reading training. The sample consisted of Dutch fourth graders with and without reading difficulties. After nine sessions of training, the authors reported that children receiving text-fading training showed significantly larger reading rate gains compared to the self-paced reading group for both proficiency levels. However, post-training assessments (measuring reading fluency on the sentence level) revealed equal gains in sentence reading fluency for both groups, indicating a lack of text-fading training specific transfer effects. Despite the robust research design, a methodological issue might have concealed possible transfer effects: A central component of text-fading training is the adaptive adjustment of text-fading rates depending on comprehension performance. While Breznitz et al. (2013) demanded more than 80% correctly answered comprehension questions before fading rates were increased; Snellings et al. (2009) increased the fading rate at thresholds of 60% comprehension accuracy. This procedure quickly led to reading rates as extreme as 10 ms per letter. It seems unlikely that fourth graders are capable of reading at these rates without comprehension loss. Indeed, the procedure resulted in fast but inaccurate reading after the fifth training session. It is likely that these fast reading rates, triggered by the training, resulted in superficial reading behavior. Hence, self-paced reading fluency gains exceeding the control group could not be detected in the post-training assessment.

To sum up, while research in adults and adolescents suggests text-fading training as an effective training tool to foster reading rate and comprehension, application of the program in children has not yet shown to be more effective than a training with self-paced reading. More precisely, the small number of previous text-fading training studies did not find differential reading fluency gains in terms of transfer effects on fluency measures after text-fading training. The current study, hence, sought to fill this gap and to investigate whether post-training silent reading fluency enhancements attributable to the text-fading training are detectable on the word, sentence and/or text level also for children with a research design comparable to Breznitz et al.'s (2013). In contrast to Snellings et al. (2009), stricter error limits were set in this study, demanding comprehension of 100% correct for an increase in text-fading rate to prevent superficial reading behavior. Contrary to Paige (2011), the used text-fading procedure was adaptive, considering the individual reading comprehension performance and also allowing for fading rate decrease if comprehension declines. Using a straight pre-post-training design, we tested whether a group of children receiving text-fading training would outperform a group reading the same material in a self-paced manner with respect to their post-training performance on a standardized reading test.

## Materials and methods

### Participants

Twenty-four German third-graders (13 females, 11 males; mean age = 9.1 years, *SD* = 0.65) from three different classes of the same school participated in the study. The school was located in a rural region of the Rhine-Main area, mainly populated by middle to high-income families. Two children were identified as outliers and later excluded from analysis, because they reached the maximum scores in both the pre- and post-test, leaving no room for improvement. Hence, 22 children were considered for data analysis (13 females, 9 males; mean age = 9.1 years, *SD* = 0.68). All children were native speakers of German. Parental informed and written consent was obtained for each child. Ethical approval was received from the local ethical review board (German Institute of International Educational Research, DIPF).

### Design

The study followed a pre-post-test design (standardized reading test measurements before and after training) with participating children assigned to one of two training groups. One group was trained with a text-fading procedure (text-fading group; *n* = 10), the other group was trained at their individual self-paced reading rate without text-fading (self-paced group; *n* = 12). In an initial assessment session, participants of both groups read 30 sentences at their own self-paced reading rate.

For the text-fading group this assessment session served to determine the individually set fading rates (in milliseconds per character) used for the subsequent text-fading training. During each of the following eight training sessions all participants read 40 sentences, depending on the group, either with a text-fading procedure (text-fading group) or without text-fading (self-paced group). Therefore, the total number of items read over the nine sessions amounted to 350 items. All sessions were distributed over a period of 3 weeks (three sessions per week) and lasted approximately 30 min each.

### Materials

#### Pre-post-test

Two parallel versions of a standardized reading test (ELFE 1-6; Lenhard and Schneider, 2006) were used to assess silent reading fluency 6 weeks before and 2 weeks after training. This test is divided into three subtests measuring reading fluency at the word, sentence and text level. According to the authors each of the three subtests shows high internal consistency as reflected by Cronbach's α of *cr*_α_ = 0.97, *cr*_α_ = 0.93, and *cr*_α_ = 0.92 at the word, sentence and text reading level, respectively.

Word reading fluency is measured through 72 items, each composed of a picture accompanied by several word alternatives, which closely resemble each other with regard to their phonemes and graphemes. Within a time limit of 3 min, children have to choose the word corresponding to the pictures. The number of correctly marked words represents the raw score for this subtest. The subtest for sentence reading fluency demands the completion of 28 sentences by choosing one of five possible word alternatives, which belong to the same word class and resemble each other in terms of graphemes and phonemes. The number of items answered correctly within 3 min constitutes the subtest's raw score. And finally, 20 items are used for measuring text reading fluency, each comprising a connected text and a corresponding multiple choice question. Again raw scores are calculated as the number of items answered correctly within a time limit of 7 min. Hence, test scores of all subtests comprise fluency measures as a combination of speed and comprehension. Conversion tables for individual raw test scores to grade-dependent standardized scores (z-scores based on a norm sample of 4893 children) are provided and were used for statistical analyses.

The test was administered as paper-pencil test in a classroom setting. All subtests consist of more items than third-graders usually accomplish in the given time frame (word reading: 72; sentence reading: 28; text reading: 20). The two children, who finished with all items correct, were therefore considered as outliers in this study.

#### Training stimuli

A pool of 350 training items adequate for third grade readers was created, each comprising a sentence (7–21 words long) and a corresponding multiple-choice question with four answer options. To prompt attentive reading sentences, questions and multiple-choice answers contained different wording and included reasonable distractors (Rost and Sparfeldt, 2007).

### Training paradigm

#### Procedure

Participants were trained in a group setting (maximum nine children simultaneously) in an extra room provided by the school. Training items were presented left-justified in black letters (font: Times New Roman; letter size: 20 pt.) against a light gray background on 15.4-inch laptops running Breznitz' original Reading Acceleration Program (Breznitz and Bloch, 2012) adapted to German, which provides the text-fading procedure. The same set and order of items was presented to all children. The initial assessment session was identical for both training groups. Every trial started with the presentation of a single sentence that the children were instructed to read as quickly and accurately as possible, once it appeared on the screen. After the children finished reading the sentence, they were instructed to click a *continue* button using the mouse, which triggered the appearance of a multiple-choice question referring to the content of the sentence. For each question, four possible answers were presented (one correct and three incorrect), and children were instructed to click on the correct answer. Once they had clicked another *continue* button, the next trial started; feedback was not provided. The average reading rate in milliseconds per character (i.e., time between sentence appearance and *continue* button-press divided by the number of characters in the respective sentence) was calculated for all correctly answered trials. After the children received instructions at the first and second session by a trained examiner, they proceeded to work independently and quietly. The examiner was present during all sessions and supervised the course of events. For the eight training sessions, the procedure differed for the two training groups.

#### Text-fading group

At the beginning of a trial, the to-be-read sentence remained visible in its entirety for 500 ms before the text-fading procedure started. This ensured that children were able to fully visually orient and begin reading the first word without interference from the fader. Individual average reading rates obtained in the assessment session served as the initial pace at which sentences disappeared from the screen letter by letter. After a sentence was fully erased from the screen, the multiple-choice question appeared. Fading rates were adjusted after blocks of five trials in a staircase-like procedure, depending on the children's reading comprehension performance: Fading rates were either accelerated by 2% (five items correct; 100% correct), kept constant (four correct; 80% correct), or decelerated by 2% (three or less correct; <80% correct). Text-fading rates in milliseconds per character as well as reading comprehension performance were recorded throughout each training session. All children started the next training session at the fading rate they had reached at the end of the preceding training session. Example movies illustrating the text fading procedure are provided in the Supplementary Material.

#### Self-paced group

The self-paced group read the same training items in the same order and responded to the same multiple-choice questions as participants of the text-fading group; however, the text-fading manipulation was not applied. Instead, the presented sentences remained on the screen until the child clicked the *continue* button, which then triggered the appearance of the multiple-choice question. Children's reading rate in milliseconds per character and reading comprehension was measured for each trial.

## Results

**Figure 1:**
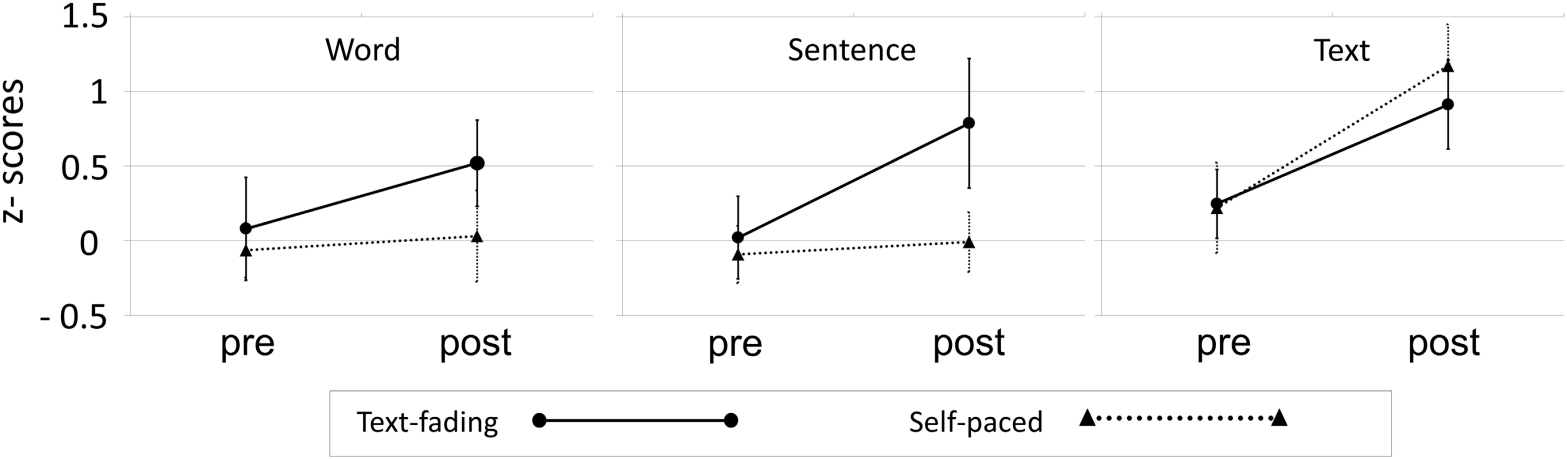
**Results of the standardized reading test**. Pre-post-training comparisons of reading fluency at the word, sentence, and text level for the two training groups.

Due to the low number of participants in each group we analyzed the data using non-parametric tests. In an initial step we controlled for baseline differences between training groups. Mann-Whitney *U*-tests for independent samples did not reveal any differences for word reading, *U* = 53.5, for sentence reading, *U* = 53.5, or for text reading, *U* = 52.0, between the two training groups in pre-tests. Furthermore, mean z-scores around zero for all three subtests (*M* = −0.04, *SD* = 0.7; see Figure 1) indicated that participants started at an average reading fluency level. Mean test scores calculated separately for both groups and time points are reported in Table 1.

**Table 1:** **Participant's test scores (raw data and standardized z-scores) for word, sentence and text reading fluency pre- and post-training for both training groups**.

	**Word reading**		**Sentence reading**		**Text reading**	
	***M***	***SD***	***M***	***SD***	***M***	***SD***
**PRE-TEST**						
Self-paced group						
raw data	44.0	8.3	19.4	3.4	13.5	3.7
z-scores	−0.06	0.64	−0.09	0.66	0.22	1.04
Text-fading group						
raw data	49.7	14.2	21.2	5.4	15.2	3.8
z-scores	0.29	1.11	0.48	1.33	0.67	1.18
**POST-TEST**						
Self-paced group						
raw data	44.2	18.2	19.8	3.5	17.2	2.9
z-scores	0.03	1.06	−0.01	0.70	1.17	0.97
Text-fading group						
raw data	54.4	12.8	23.5	5.2	17.1	3.3
z-scores	0.69	0.92	1.12	1.46	1.22	1.12

*SD, standard deviation; M, mean; presented z-scores are standardized scores based on a norm sample of 4893 children; maximum raw test score for word reading = 72, for sentence reading = 28, for text reading = 20*.

**Figure 2:**
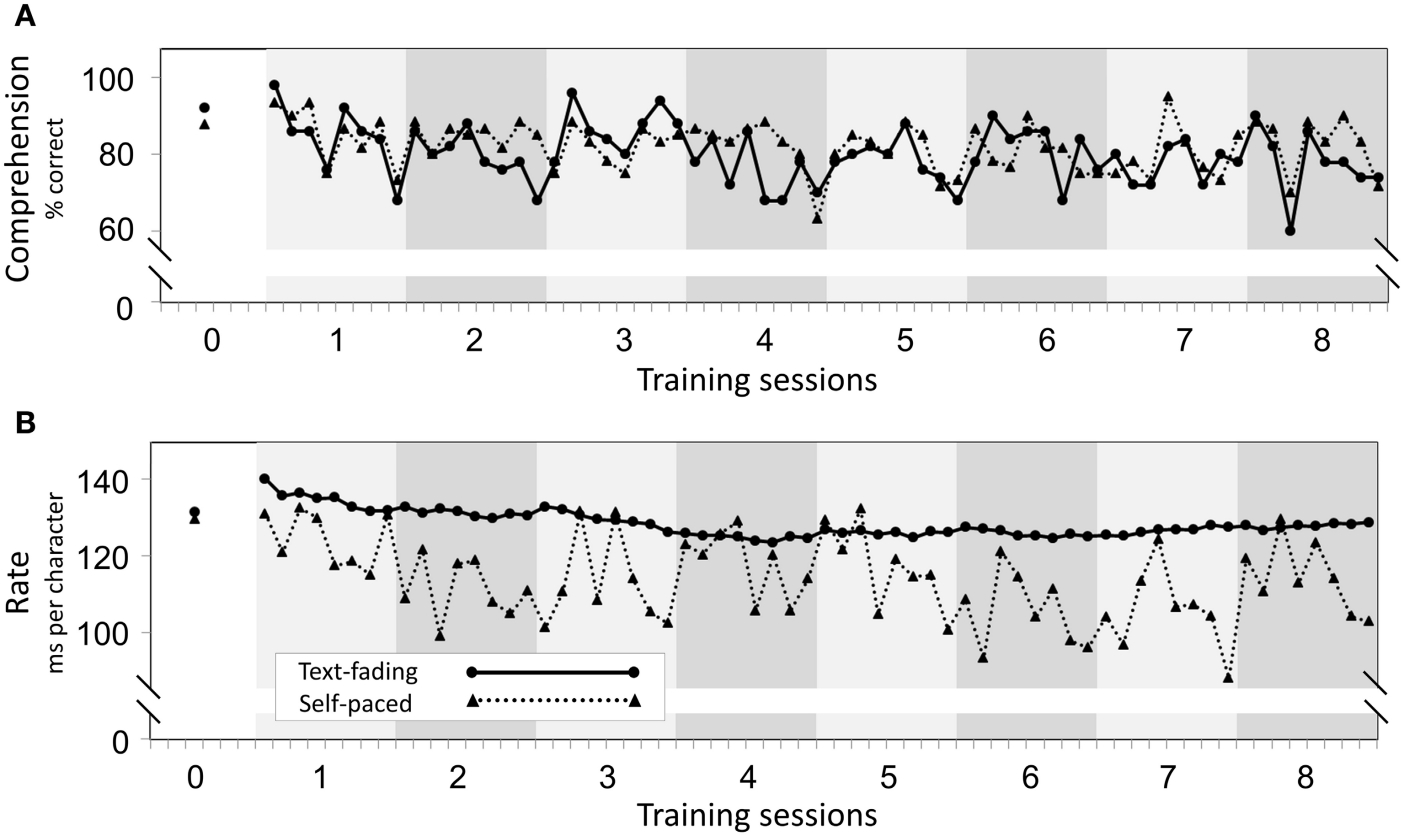
**Training time course. (A)** Illustrates the time course of reading comprehension for the two training groups. **(B)** Depicts the time course of reading rate for the self-paced group and fading rate for the text-fading group. Reading rate represents the time between the sentence presentation and the continue button-press divided by the number of characters of the respective sentence. Fading rate was calculated as the time duration between the presentation of a sentence until the last letter was erased (including the 500 ms delay) divided by the number of characters. Each tick on the x-axis represents the average of five reading items. Training session 0 refers to the initial assessment session at which a total of 30 reading items were read to measure the individual reading rate per character.

For the self-paced group, the rate measure reflected the actual reading speed. For the text-fading group, however, the text-fading rate only specified the rate at which letters disappeared from the screen and thus did not reflect the actual reading rate. A direct comparison of reading and fading rates between groups would therefore not be appropriate, and we abstained from conducting such statistical analyses. However, for informational purposes, the development of reading rate, text-fading rate, and reading comprehension over all training sessions is illustrated in Figure 2.

We performed Wilcoxon signed-rank test for paired samples to test effects of Time (pre- vs. post-test). Tests were separately conducted for each Group (text-fading vs. self-paced). While word reading fluency improved for the text-fading group, *Z* = 2.25, *p* = 0.024, the difference for the self-paced group was not significant, *Z* = 1.14, *p* = 0.255. Similar results were found for sentence reading; while the text-fading group performed better after training, *Z* = 2.67, *p* = 0.008, no change in sentence reading fluency was detected for the self-paced group, *Z* = 0.71, *p* = 0.477. Text reading fluency improved significantly for both the text-fading group, *Z* = 2.55, *p* = 0.011, and the self-paced group, *Z* = 2.39 *p* = 0.017.

These separately conducted analyses suggest an interaction of the factors Time and Group, however, they do not reveal interaction effects. Non-parametric procedures that allow the estimation of interaction effects are still not widely used. However, in order to test for interaction effects we opted for presenting the results of the aligned-rank transform procedure (ART) developed in 1994 by Higgins and Tashtoush (as cited in Nussbaum, 2015) in addition to the Wilcoxon signed-rank tests presented above. The ART extends rank transformation procedures that were shown to be unsuitable for the detection of interactions in a manner that main effects are first subtracted from raw data and second rank-transformed before an analyses of variance (ANOVA) is conducted. The removal of the main effect thus isolates the interaction effect. After eliminating the main effect of Time and rank-transforming our raw data we conducted repeated measures ANOVAs, which yielded a significant interaction of Time × Group for sentence reading fluency, *F*_(1, 20)_ = 8.92, *p* = 0.007. However, no significant interactions were found for word, *F*_(1, 20)_ = 1.13, *p* = 0.301, and text reading fluency *F*_(1, 20)_ = 0.67, *p* = 0.424.

## Discussion

In this study, we investigated for a group of German third-graders if silent reading fluency on the word, sentence and text level can be improved through text-fading training. In contrast to previous studies using text-fading training in samples of children (Snellings et al., 2009; Paige, 2011; Berninger et al., 2013), this study found significantly larger post-training sentence reading fluency gains in a standardized reading test for a group receiving text-fading training compared to a group reading at their self-paced reading routine. These gain advantages indicate that sentence reading fluency was significantly enhanced as a result of the text-fading training. Hence, text-fading training seems to represent a suitable tool for the improvement of sentence reading fluency also for children.

It is conceivable that the detection of these effects was facilitated by the modified study design: we strictly focused on silent reading in a homogeneous sample (in terms of age and reading fluency) and compared text-fading to self-paced reading training. But most importantly, in contrast to a previous study with Dutch children (Snellings et al., 2009), we adjusted text-fading rates more cautiously, which ensured that comprehension was not impaired by extreme text-fading rates. However, a generalization of our findings to other age or proficiency groups does not seem appropriate. Whether dyslexic readers, who, more than normally achieving children, are in need of intervention, can profit from transfer effects attributable to text-fading training will need to be clarified in future studies.

The question remains why differential transfer effects were only observed for sentence reading fluency. Obviously, participants were trained in sentence reading; it is therefore not surprising that effects were strongest at this level. Results at the word level showed a significant improvement for the text-fading group and no effect for the self-paced group in the Wilcoxon signed-rank test; the ART, however, did not find a significant interaction effect. Hence, the findings on the word level have to be interpreted with caution. It can be argued that the text-fading group improved on the word level since fast word recognition represents the basis for fast sentence reading. Nevertheless, future research studies will have to clarify whether differential effects with significant interactions between groups can also be revealed on the word reading level.

Text reading was not explicitly trained in this study. However, both groups improved equally and did not differ statistically as no interaction effect was revealed for text reading fluency. As outlined earlier, text reading requires a large variety of skills and is therefore highly challenging for beginning readers. Compared to single word reading, which primarily demands proficient decoding (Klauda and Guthrie, 2008) as well as skills that are generally required for successful reading performance (such as memory and attention; Johnston and Kirby, 2006; Primor et al., 2011), connected text reading requires additional skills (such as semantic skills, the use of linguistic context as well as background knowledge; Rapp and van den Broek, 2005; Florit et al., 2013) for successful processing. Consequently, improvements in text reading comprehension may be attributed to a multitude of different factors. Furthermore, it could be that gains in text reading were driven by a focus on text processing in the school's curriculum during the study period, which may have resulted in an improved text reading fluency for both groups. Therefore, it might be that specific training effects on text reading level were concealed by other factors influencing text reading fluency. As the here presented training was based on isolated sentence reading, it seems worth investigating whether text level training may produce similar effects as a sentence based training. If other factors influencing text reading comprehension could be controlled for, it might be possible to also reveal differential reading fluency gains through text-fading training at text level. Considering that text reading is of particular importance in the educational system and for successful reading acquisition, such performance gains would be highly valuable for directed and self-directed learning and should therefore be the focus in future studies.

Certainly, there are several limitations. The small sample size and the relatively small number of training trials are limiting the statistical power of this study. A larger sample size would have provided more robust data and might have allowed the differentiation of subsamples with varying reading proficiency levels. Furthermore, as outlined in a recent meta-analysis comparing reading training approaches (Galuschka et al., 2014), it seems advisable to design training studies lasting more than 12 weeks to reveal a training's full potential and effectiveness. Hence, if more training sessions would have been integrated in this study design, reading fluency improvements might have been even stronger. An additional limitation of the study is that the implemented reading material was not experimentally varied. Given that reading material characteristics (e.g., word or sentence length, word frequency, categories) have a direct impact on reading fluency (Hiebert and Fisher, 2010; Nagler et al., 2014), reading material specific training effects should be in the focus of future text-fading training studies.

Nonetheless, the present study followed the National Reading Panel's (2000) demand to engage in the investigation of silent reading fluency trainings. The presented results are encouraging as they suggest that text-fading training can be effective to improve children's silent sentence reading fluency. Furthermore, text-fading training has a number of economic advantages (e.g., its applicability in classroom settings demanding a minimum of teacher supervision). Hence, a logical next step of testing the program's efficacy and efficiency would be the comparison with already established reading training approaches. As described above, previous studies (Paige, 2011; Berninger et al., 2013) focusing on this comparison were not able to prove text-fading training to be more effective than existing training methods. However, considering the studies research designs it might have been a number of experimental design issues that have prevented the detection of text-fading specific training effects: Berninger et al. (2013) embedded their text-fading training study within a battery of training approaches, which makes it difficult to isolate the distinct contribution of text-fading training to the overall training gain. Additionally, their participants' high variance in terms of age (9–15 years) might have concealed effects of age specific training responsiveness. Paige (2011) employed a rather arbitrary way of increasing the text-fading rate (i.e., a non-adaptive and fixed text-fading rate increase), disregarding comprehension performance. It is therefore not directly comparable to text-fading training. Furthermore, the reading proficiency level of the participating sample was not controlled for (i.e., classification relied on external diagnoses) and was presumably highly diverse. Hence, to investigate the efficacy of text-fading training and potential advantages, a well-designed study needs to be conducted, comparing text-fading training to established reading training programs, such as for instance the repeated reading approach.

Although it was neither in the scope nor the aim of the current study to investigate the underlying cognitive factors responsible for the observed training gains, it would be desirable to know the active principle of text-fading training. The investigation of possible underlying factors might allow for a more targeted assignment of children in need to this specific approach. Even though some theoretical assumptions have been made, such as for instance a reduction of working memory load, suppression of phonological recoding or improved attention allocation (Breznitz, 1987, 1988, 1997a,b; Breznitz and Share, 1992), a comprehensive examination determining the causes for improvements of reading fluency is still due. Additionally, the influence of text-fading training onto children's decoding strategy behavior should be investigated in more detail to examine the assumption that time constraint may induce strategy shifts from lower to higher elaborated strategies (i.e., from procedural-related to retrieval-related strategies).

In summary, we were able to detect differential transfer effects on sentence reading fluency as a result of text-fading training. Text-fading based reading training therefore seems to represent an effective, ecologically valid, and economically attractive training approach for improving silent sentence reading fluency not only in adults, but also in children. However, further research will have to investigate the training's applicability for different reading proficiency levels and age groups, as well as for its effectiveness on word and text reading level. Furthermore, text-fading training should be compared to established reading training programs to estimate its efficacy, and underlying cognitive factors as well as reading strategy behavior possibly accountable for improvements should be investigated in detail.

### Conflict of interest statement

The authors declare that the research was conducted in the absence of any commercial or financial relationships that could be construed as a potential conflict of interest.
