# Supplementary material for: Text-fading based training leads to transfer effects on children's sentence reading fluency
Source: Front Psychol. 2015 Feb 10;6:119. doi: 10.3389/fpsyg.2015.00119 (PMC4322541; doi:10.3389/fpsyg.2015.00119)
Supplement: Supplementary file 3 [file DataSheet1.DOCX]

***Supplementary Material***

**Text-fading based training leads to transfer effects on children’s sentence reading performance**

**Telse Nagler^1,2^*, Sebastian Peter Korinth^2,3^, Janosch Linkersdörfer^1,2^, Jan Lonnemann^1,2^, Björn Rump^1,2^, Marcus Hasselhorn^1,2,3^, Sven Lindberg^1,2^**

^1^German Institute for International Educational Research (DIPF), Frankfurt am Main, Germany
^2^Center for Individual Development and Adaptive Education of Children at Risk (IDeA), Frankfurt am Main, Germany

^3^Department of Psychology, Goethe University Frankfurt am Main, Frankfurt am Main, Germany

*** Correspondence:** Dr. Telse Nagler, German Institute for International Educational Research (DIPF), Schloßstraße 29, 60486 Frankfurt am Main, Germany.

nagler@dipf.de

1. **Supplementary Data**

**Video 1.** Video sequence exemplarily illustrating the text-fading manipulation at the beginning of the text-fading training for a slow reading child.

**Video 2.** Video sequence exemplarily illustrating the text-fading manipulation in a progressed training session with accelerated text-fading rate.
